# Case report: multiple gastrointestinal stroma tumors in the background of neurofibromatosis type 1

**DOI:** 10.1093/jscr/rjaa516

**Published:** 2020-12-24

**Authors:** Tim N Beck, Kevin Brown, James Lapinski, Ram K Gurajala, Kasra Karamlou, Toms Augustin

**Affiliations:** Department of General Surgery, Cleveland Clinic, Cleveland, OH, USA; Department of General Surgery, Cleveland Clinic, Cleveland, OH, USA; Department of Pathology, Cleveland Clinic, Cleveland, OH, USA; Department of Diagnostic Radiology, Cleveland Clinic, Cleveland, OH, USA; Department of Hematology and Medical Oncology, Cleveland Clinic, Cleveland, OH, USA; Department of General Surgery, Cleveland Clinic, Cleveland, OH, USA

## Abstract

Gastrointestinal stromal tumors (GISTs) are the most common mesenchymal malignancies of the gastrointestinal tract. GISTs can occur in the background of neurofibromatosis 1 (NF-1), where chemotherapeutic treatment is not optimal and surgical intervention is the only management option. In this case report, we present a case involving a 61-year-old gentleman with NF-1. The patient presented with acute blood loss anemia that was initially controlled with embolization of a hyper-vascular mass abutting the distal jejunum. The patient was taken to the operating room for excision of the mass. All macroscopic disease was excised and the pathology noted GISTs. Surgical decision making is not clearly delineated in the literature for GISTs in patients with NF-1, where targeted therapy is not a treatment option. Resection of all disease should be considered, since NF-1 associated GISTs generally do not have harbor mutations that can be targeted.

## BACKGROUND

Gastrointestinal stromal tumors (GISTs) are the most common mesenchymal malignancies of the gastrointestinal tract and originate from the interstitial cells of Cajal [1]. Incidence of GIST is estimated around 14.5 per million [1], with the most commonly affected anatomic sites being the stomach (60%) and the small intestine (30%). The duodenum is rarely involved (<5%) and the same goes for the colon and rectum (<5%). Metastatic disease is rare and generally involves the liver, bones and lung. The 5-year survival for GIST is 94%, 82% and 52% for contained, locally advanced and metastatic disease, respectively [2].

Sporadic mutations are the dominant genetic driver of GIST formation. The most commonly mutated gene in GIST is *c-KIT*. Additional common sporadic GIST mutations involve the platelet-derived growth factor receptor alpha (*PDGFRAα*) gene. Together, *c-KIT* and *PDGFR* gain-of-function mutations are involved in ~85% of GISTs [3].

GIST has also been linked to hereditary diseases, particularly Carney Triad, Carney-Stratakis syndrome and neurofibromatosis type 1 (NF-1 or von Recklinghausen’s disease). NF-1 is an autosomal dominant disorder occurring in 1 of 3000 births and is characterized by multiple cutaneous neurofibromas, café-au-lait spots, freckling and Lisch nodules. In NF-1, the tumor suppressor gene *NF-1* is mutated, resulting in reduced production or loss of function of the protein neurofibromin. Neurofibromin is a suppressor of Ras activity via hydrolysis of GTP bound to active Ras. In the setting of mutated *NF-1*, increased Ras activity is observed [4], resulting in a similar cellular response seen in *c-KIT*-mutated GIST.

In terms of GIST management, surgery is the only curative modality and even in the metastatic or locally advanced disease setting, cytoreductive surgery is sometimes cited as beneficial [5–7]. The surgical approach to multifocal disease is less clear, although a majority of reports suggest favorable outcomes with surgical treatment in such patients.

In this case report, we present a case of urgent surgery for a bleeding jejunal mass in a patient with NF-1. We here discuss the critical decision making for a complex surgical case of this nature.

**Figure 1:**
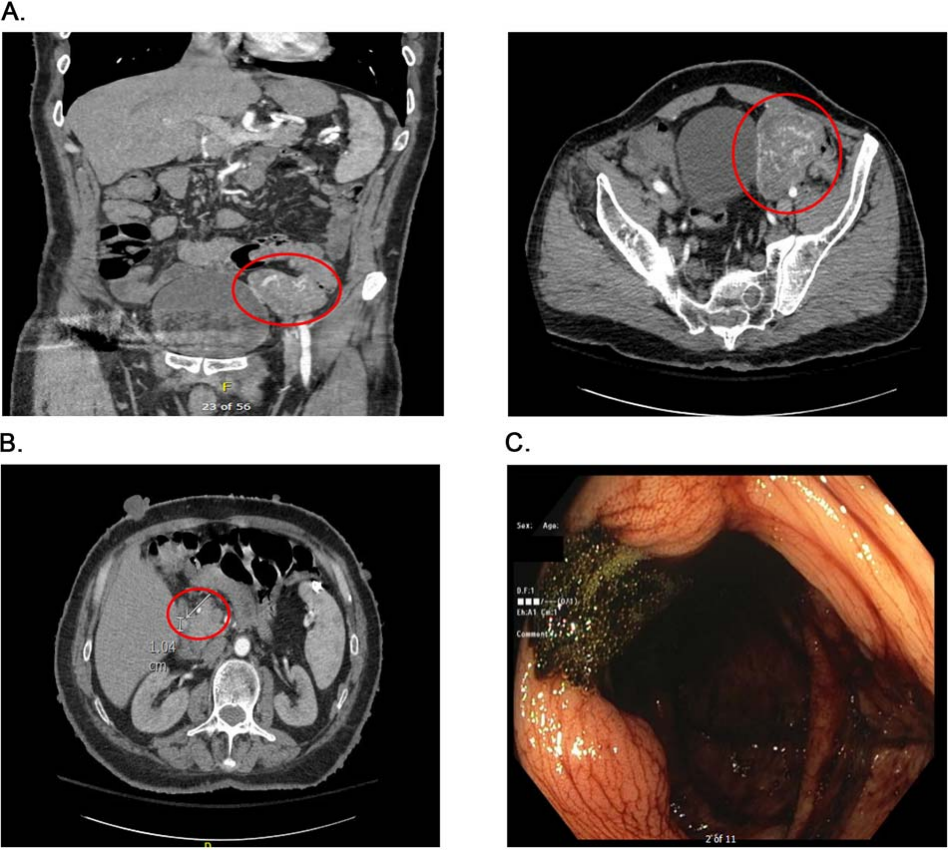
Presurgical diagnostics. (**A**) CT imaging of the large jejunal mass. (**B**) CT imaging of small duodenal mass. (**C**) Blood at the ileo-cecal valve.

## CASE PRESENTATION

The patient is a 61-year-old male with a history significant for NF-1, hypertension, esophagitis and intermittent gastrointestinal bleeding, who initially presented to an outside institution for acute onset of bleeding per rectum. The patient noted bloody bowel movements with diarrhea for several days, which had increased in frequency over the past 24 hours. The patient reported mild abdominal pain and denied nausea, emesis, chest pain, shortness of breath, syncope or other symptoms at the time. He was not on anticoagulation and denied excessive alcohol or nonsteroidal anti-inflammatory drug (NSAID) use. His family history was significant for NF-1 diagnosed in his mother and brother. He had no significant surgical history other than a total hip replacement.

On admission at the outside hospital, the patient’s hemoglobin was 6.6 g/dl, with normal platelets, partial thromboplastin time and prothrombin time. Diagnostic workup at the outside institution included a nuclear medicine gastrointestinal scan (mTc-labeled RBCs), which was negative for abnormal bleeding. On hospital Day 2, his hemoglobin failed to respond appropriately to transfusions and a computed tomography angiogram (CTA) was completed, which showed a 6.9 × 5.8 × 6.8 cm hyper-vascular mass in the left pelvis; abutting the left border of the bladder medially, iliac arteries and veins posteriorly, rectus sheath anteriorly and the small and large bowel (Fig. 1A). Vascular supply of the mass was identified to originate from branches of the superior mesenteric artery (SMA) and appeared to be contiguous with the adjacent jejunum. The mass was noted to also abut, but not involve, the sigmoid colon. The CT scan further noted a 9 mm enhancing tumor in the second portion of the duodenum anteriorly (Fig. 1B). After administration of five total units of packed red blood cells with limited improvement in hemoglobin, the patient was transferred to our institutions for further management.

**Figure 2:**
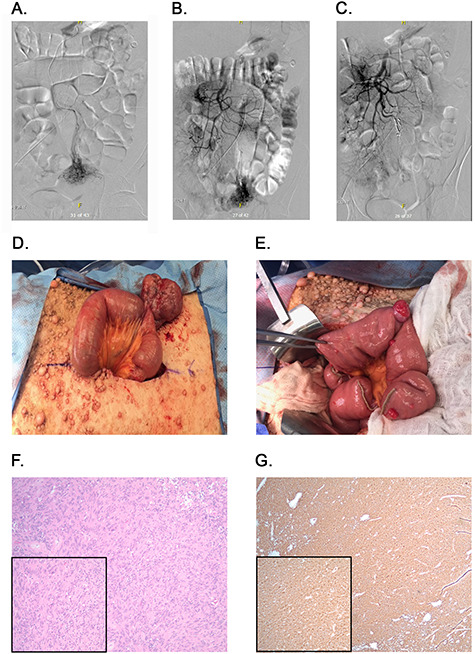
Angiography, embolization and macroscopic and microscopic views of the tumors. (**A**) Angiography identifying blood flow to the mass. (**B**) Blood flow from ileal branches of the SMA to the jejunal mass. (**C**) Embolization of the blood supply to the jejunal mass. (**D**) Large jejunal mass. (**E**) Stretch of proximal jejunum with multiple masses. (**F**) H&E stained tumor slides; ×100 magnification with inset ×200 magnification. (**G**) DOG-1 stained tumor slides; ×40 magnification with inset ×100 magnification.

The patient arrived hemodynamically stable and underwent esophagogastroduodenoscopy (EGD). The EGD was unremarkable with no visualization of the previously mentioned duodenal mass. Colonoscopy was performed, which was notable for bright red blood present throughout the entire colon as well as blood 5 cm proximal to the ileocecal valve (Fig. 1C). As the patient continued to have bloody output per rectum, with insufficient response to transfusion, interventional radiology was consulted and proceeded with mesenteric angiography with intervention (Fig. 2A). The hyper-vascular mass was found to be supplied by ileal branches of the SMA, which were successfully embolized (Fig. 2B and C). After conversations with the interventional radiology team and the patient, the decision was made to proceed with an exploratory laparotomy and resection of the identified mass.

Exploratory laparotomy was notable for a 7 cm mass originating from the distal jejunum (Fig. 2D). This mass was mostly exophytic and a single firing of a GIA load was used to excise the mass with preserved adjacent normal wall of the jejunum. Kocherization of the duodenum revealed a single sub-centimeter duodenal mass at D2. The mass was excised with subsequent primary closure of the defect. Taking down the Ligament of Treitz allowed us to identify two additional proximal jejunal lesions that were resected and resulting defects were primarily closed. The remainder of the jejunum was evaluated and a stretch of proximal jejunum, about 10 cm from Treitz, containing 10 small (0.5 to 2 cm) lesions was identified (Fig. 2E). The decision was made to resect a 30 cm segment of jejunum with creation of a side-to-side functional end-to-end anastomosis using a linear stapler. This allowed complete resection of macroscopic disease. The mesenteric defect was closed using multiple interrupted silk sutures. The remainder of the bowel was examined and found to be normal. The patient returned to the intensive care unit and recovered over the course of the coming days without immediate complications and without requiring additional transfusions.

Five surgical specimens were sent to pathology, including the large intra-abdominal mass, a segment of proximal jejunum (30 cm), the duodenal lesion and two proximal jejunal lesions. Microscopic examination revealed a total of 13 foci of tumor. All tumor margins were negative and 12 regional lymph nodes were examined and revealed no evidence of nodal metastases. All tumors underwent H&E staining (Fig. 2F) and immunohistochemical staining for DOG-1 (Fig. 2G), which was strongly positive, confirming a diagnosis of GIST for all tumors.

Genetic analysis of the tumors samples was performed on paraffin-embedded tissue using the Tempus XT next generation sequencing (NGS) platform, which includes 648 cancer-related genes. No *KIT* or *PDGFRA* mutations were detected (both were wild type). Instead, *NF-1* was found to harbor a loss-of-function single nucleotide alteration (c.654 + 1G > T splice region variant).

The patient recovered appropriately from his surgery and was discharged one week after the operation. He continues to do well and has returned to full-time work.

## DISCUSSION AND CONCLUSION

Surgery remains the only curative intervention for GISTs and in the metastatic setting, cytoreductive surgery may provide a survival advantage [3, 5, 8, 9]. The reported case is unlikely to represent metastatic disease; rather, the tumors all appear to be primary tumors, as previously reported in patients with germline *NF-1* mutation.

The correlation between GIST and NF-1 is well established and in a longitudinal study of 70 patients with NF-1, 7% were found to develop intestinal sarcoma [10]: a significantly higher rate than is observed in the general population. GISTs in the context of NF-1 are often indolent, asymptomatic and with low mitotic rates, as was seen in our case. *c-KIT* and *PDGFR* are only mutated in about 4.8% of GIST in NF-1 cases.

Tumor location is an important factor to consider for surgical management and prognosis. For duodenal lesions, local excision is recommended if feasible, given the significantly higher complication rate for pancreaticoduodnectomy. Pancreaticoduodenectomy may be required for large duodenal GISTs (>5 cm) and GISTs proximal to the ampulla of Vater. Duodenal tumors <2 cm in size generally have low aggressive behavior and can be followed with periodic surveillance or can be resected with primary closure. In cases of resection, R0 margin is the goal. Additionally, complete resection of all visible disease is the desired outcome in general [8].

In the described case, an intervention was urgently indicated given the patient’s significant bleeding. CT-angiography is generally sufficient to prompt laparotomy. EGD and colonoscopy were performed to rule out alternative sources for the bleeding, given that CT-angiography and mTc-labeled RBCs did not definitively show active bleeding, although the vascular mass was clearly identified on CTA. IR embolization allowed time for optimal resuscitation of the patient and proper planning for surgical intervention that might have included extensive resection based on imaging findings.

Our case highlights the challenge of surgical management of patients with multiple GISTs and raises awareness of the possible presence of multiple GISTs in the setting of *NF-1* mutation. This case also highlights the absence of usual molecular drivers in patients with GIST in the setting of aberrant *NF-1*. These tumors have a different clinical and biological pattern of behavior. Due to lack of molecular targets, *NF-1* mutated GISTs are less likely to respond to standard medical management in the adjuvant, neo-adjuvant and metastatic setting. Currently, oncological resection is the goal of therapy with vigilant postoperative surveillance to detect any new tumors that may arise.

## CONFLICT OF INTEREST STATEMENT

None declared.

## FUNDING

No funding or grant support.
